# Estimation of Mental Effort in Learning Visual Search by Measuring Pupil Response

**DOI:** 10.1371/journal.pone.0021973

**Published:** 2011-07-08

**Authors:** Tatsuto Takeuchi, Théodore Puntous, Anup Tuladhar, Sanae Yoshimoto, Aya Shirama

**Affiliations:** 1 Department of Psychology, Japan Women's University, Kawasaki, Japan; 2 LTDS, CNRS UMR 5513, Université de Lyon, ENISE, Saint-Etienne, France; 3 Department of Chemical Engineering, University of Waterloo, Waterloo, Ontario, Canada; 4 Perception and Emotion Research Group, NTT Communication Science Laboratories, Atsugi, Japan; 5 Japan Science and Technology Agency, Core Research for Evolutional Science and Technology (JST CREST), Tokyo, Japan; University of Sydney, Australia

## Abstract

Perceptual learning refers to the improvement of perceptual sensitivity and performance with training. In this study, we examined whether learning is accompanied by a release from mental effort on the task, leading to automatization of the learned task. For this purpose, we had subjects conduct a visual search for a target, defined by a combination of orientation and spatial frequency, while we monitored their pupil size. It is well known that pupil size reflects the strength of mental effort invested in a task. We found that pupil size increased rapidly as the learning proceeded in the early phase of training and decreased at the later phase to a level half of its maximum value. This result does not support the simple automatization hypothesis. Instead, it suggests that the mental effort and behavioral performance reflect different aspects of perceptual learning. Further, mental effort would be continued to be invested to maintain good performance at a later stage of training.

## Introduction

Learning is a lifelong endeavor. Different learning processes such as explicit ones for memorizing things, events, and locations or implicit ones that proceed in an unconscious manner, are known to be functioning concurrently [1]. In this study we estimated subjects' mental effort (or mental load) invested in the process of perceptual learning, one of the implicit learning processes. In visual perceptual learning, improvement of perceptual sensitivity or behavioral performance is observed after subjects have extensively trained for a specific visual task [2]–[4].

An interesting hypothesis regarding learning is that the behavior of the subjects becomes “automatic” as learning proceeds [5] [6]. Thus, the task can be accomplished easily after intensive learning. One example of this automaticity is driving a car daily between home and workplace. Even though driving is a highly complex behavior, most people do not even remember how they drove home yesterday. A specific task, which would be very difficult for a naïve subject, is sufficiently accomplished without allocation of an attentional resource, called mental effort after learning.

In this study, we examined how the amount of invested mental effort varies as perceptual learning proceeds: Does mental effort linearly decrease as behavioral performance for the learned task increases in the course of training? Or is the estimated “learning curve” of mental effort different from that of the behavioral performance? Another possibility is that to maintain better performance through the training, subjects' invested mental effort continues to increase, as indicated by Leonards et al [7]. We attempted to answer these questions by measuring pupil size of subjects while they conducted a visual search task.

The strength of responses of the autonomic nervous system has been considered to reflect the amount of invested mental effort [8] [9]. Pupil response is governed by the autonomic nervous system. Pupil dilation depends on the activation of the adrenergic sympathetic nervous system, while pupil constriction depends on the cholinergic parasympathetic nervous system [10]. It has been well documented that pupil response is modulated not only by an ambient luminance level (the so-called pupil light reflex) but also by the amount of mental effort invested in a task [11]–[18]. For example, Porter et al [16] reported that the pupil dilates when subjects conduct a difficult visual search task in which high mental effort has to be invested.

**Figure 1:**
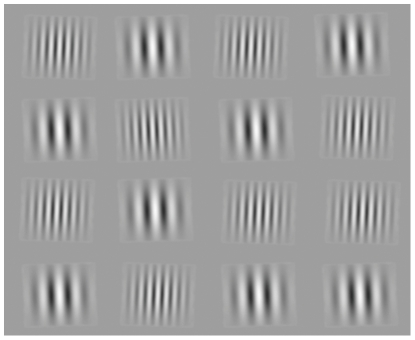
Typical display for the visual search task. Sixteen Gabor patches appeared on the screen. Half of the trials contained a target, which was defined by the combination of orientation and spatial frequency. In this display, the target has a high spatial frequency and is oriented counter-clockwise. It is located at second-row and second-column. The position of the target and the distracters were randomized between trials.

In this study, we had subjects conduct the so-called conjunction visual search task [19] while we measured their pupil diameter with an infrared-video-based eye-tracking device. Figure 1 shows a typical display for the conjunction search task. The display contains one target, defined by the combination of orientation and spatial frequency of a Gabor patch. The task was for the subjects to report whether the target existed or not in the display as quickly as possible while maintaining accuracy. Reaction time was measured by tapping a sensitive touch-pad to minimize body movement. Set size was fixed to 16. Throughout all sessions, half of the trials contained a target; the other half did not. As shown in Fig. 1, the conjunction search task used in this study is basically difficult, and the subjects had to search the target in a serial manner [19] [20]. By using a difficult search task, we expected to be able to observe both the learning effect and pupil size change depending mental effort.

On each day, subjects conducted 64 trials (half of which contained a target) of the visual search task. Subjects continued the experiment for 16 consecutive days. We predicted that if the learning decreases mental effort, as experienced in daily driving, then pupil size would decrease as the learning proceeds. On the other hand, Leonards et al. [7] showed that the skin conductance response, an autonomic response solely governed by the sympathetic nervous system [21] [22], increases as the reaction time decreases in the course of visual search training. They argued that a difficult parallel visual search task never becomes “automatic” and that the amount of invested mental effort never decreases. If this argument could be applied to different autonomic nervous responses, we predicted we would observe an increase in pupil size as the learning proceeded in our study.

## Results

**Figure 2:**
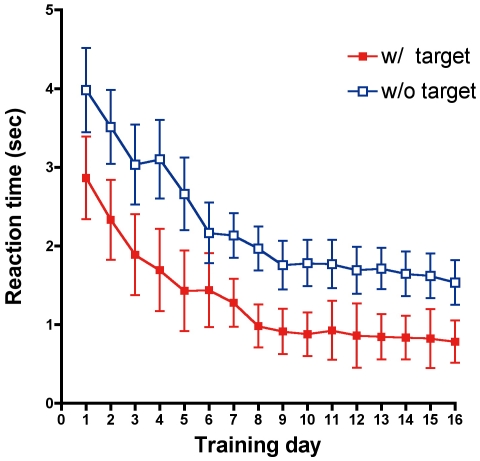
Learning curve of the conjunction search task. Reaction time in seconds as a function of training day is plotted. Filled red squares denote the data when the target is presented. Blank blue squares denote when the target is absent. The data from 12 subjects are averaged. Error bars represent ±1SEM.

The learning dynamics of the conjunction search task over the entire training period is shown in Fig. 2. Each data point represents the average of 32 trials per subject. As the training proceeded, the reaction time significantly decreased to the asymptote at around 1 sec when the target was present. When the target was absent, the reaction time significantly decreased from about 4 sec on the first day to less than 2 sec in the late training phase. A two-way ANOVA shows that the main effect of training day and task (target-present or -absent) were significant (F(15, 165) = 6.35, p<0.0001 for the training day; F(1, 11) = 49.02, p<0.0001 for the task). No interaction between training day and task was found (F(15, 165) = 0.13, n.s.). A similar learning curve has been reported in the visual search literature [23]–[25].

Subjects received feedback regarding the error rate after each session (One session consisted of eight trials. Each subject completed eight sessions per day), and they were instructed to try to reduce the error rate if it was high. The average error rate for all subjects decreased from 8.2% on the first day of training to 1.6% on the last day. Thus, the decreasing reaction time in Fig. 2 is not a by-product of speed accuracy trade-off. We observed 99.5% of the error for trials in which a target was present. Since errors were quite rare in the target-absent trials, we did not use the error rate in the following data analysis.

We evaluated the pupil diameter only in target-absent trials for three reasons. First, the target-absent trials were almost completely error free as described above. Second, it can be assumed that subjects observed each Gabor patch in the target-absent trials. The ratio of the reaction time between target-present trials and target-absent trials was 1.96 on the last day of the training (Fig. 2), and no interaction between the task and the training day was found as described above. This suggests that the observers conducted the visual search task in a typical serial manner [19] [20]. Third, we found in our preliminary observation that pupil size sharply increased when subjects found the target. This kind of orienting response was not observed in the target-absent trials. On the basis of those three points, we assumed that the pupil data from the target-absent trials would be more stable than that in the target-present trials, and therefore concentrated our analysis on the target-absent trails. However, it should be noted that we confirmed that the same tendency was observed both in target-present and target-absent trials, though the variance is smaller in the latter.

**Figure 3:**
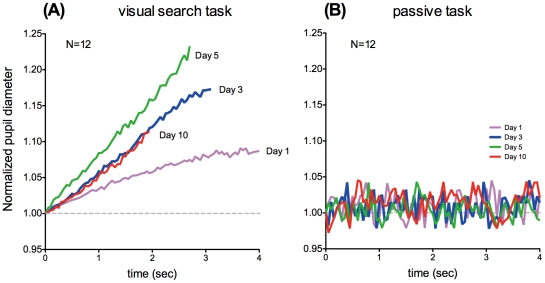
Normalized pupil diameter as a function of time. (A) Each function represents the averaged pupil diameter of 12 subjects during the conduction of the visual conjunction search task. The data in each trial was normalized relative to the pupil size during the cross fixation period immediately preceding the trial. Different functions denote the different training day: 1^st^, 3^rd^, 5^th^, and 10^th^ day, respectively. (B) Each function represents the averaged pupil diameter of 12 subjects conducting the passive viewing task. In passive condition subjects observed the visual search display, but no task was imposed. The data in each trial was normalized relative to the pupil size during the cross fixation period immediately preceding the trial. Different functions denote the different experimental day: 1^st^, 3^rd^, 5^th^, and 10^th^ day, respectively.

Figure 3(A) shows normalized time-varying pupil size in target-absent trials on the 1^st^, 3^rd^, 5^th^ and 10^th^ day of training for all subjects. A visual search display was presented at 0 sec. Eye blinks were removed by standard spline interpolation. As shown in the graph, pupil size monotonically increased as the subjects started to search for a target. This tendency was observed independent of the training day. However, the rate of increase was varied between different training days. It was slowest at Day 1, and gradually increased to a maximum at Day 5. At Day 10, it was slower than at Day 5 and was similar to that at Day 3. The differences in the rate of increase of the functions shown in Fig. 3(A) indicate that the pupil size varied as the learning of visual search proceeded. Pupil size became larger at the early phase of learning and started to decrease once it reached a maximum at Day 5.

**Figure 4 pone-0021973-g004:**
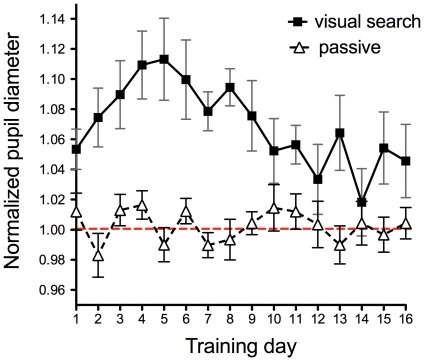
Normalized average pupil diameter as a function of the training day. Filled squares denote the averaged pupil diameter of 12 subjects conducting the visual conjunction search task. In the passive condition (blank triangles), subjects observed the same display but no task was imposed. The data was calculated from Fig. 3. Error bars represent ±1SEM.

To quantitatively evaluate pupil size during the visual search task, the average diameter of the pupil from the start of the task to the subjects' response was calculated from Fig. 3(A). Figure 4 shows the averaged pupil diameter as a function of training day. A one-way ANOVA shows the main effect of training day on pupil size (F(15, 165) = 13.6, p<0.0001). The data show that pupil diameter monotonically increased during the early phase of training, reached a maximum at the 5th day of the training, and then decreased to a level half of the maximum at the late phase of learning. Thus, it can be said that the shape of the pupil size function is quite different from the behavioral performance, in which the reaction time decreases monotonically as shown in Fig. 2.

**Figure 5:**
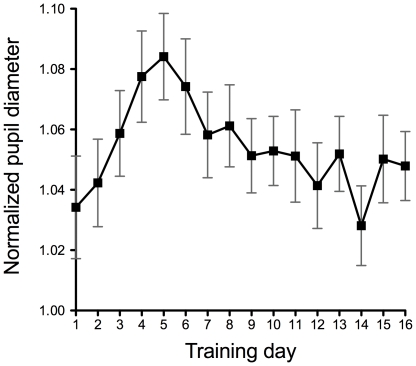
Normalized pupil diameter 1 second after the onset of the visual search display. Filled squares denote the averaged pupil diameter of 12 subjects 1 second after the onset of the visual search display, as a function of the training day. The data was calculated from Fig. 3(A). Error bars represent ±1SEM.

In Figure 5, we plot the normalized pupil size measured at 1 sec after the onset of the visual search display. This data reflects the rate of increase in pupil size as a function of the training day. It increased during the early phase of training, reached a maximum on the 5th day of the training, and decreased to a level half of the maximum in the late phase of learning. A one-way ANOVA shows the main effect of training day on pupil size (F(15, 165) = 11.2, p<0.0001). The close similarity between Fig. 4 and Fig. 5 indicates that the learning of visual search modulated the rate of increase in pupil size, which induced an increase and decrease in the averaged pupil size while learning visual search.

One question is whether the pupil size only reflects the mental effort by the subjects or the pupil light reflex might contaminate the results. To confirm this we ran a “passive” viewing condition in which subjects were asked to observe the visual search display in a relaxed manner, in which searching for the target and responding were not required. Figure 3(B) shows normalized time-varying pupil size in the passive viewing condition, lasting for 4 sec, on the 1^st^, 3^rd^, 5^th^ and 10^th^ experimental day for all subjects. A visual search display was presented at 0 sec. As shown in the figure, the pupil size did not increase or decrease significantly after the onset of the visual search display in the passive viewing condition. Blank triangles in Figure 4 show the averaged pupil diameter as a function of day in the passive condition, calculated from Fig. 3(B). The pupil diameter is normalized by the diameter during the cross fixation period immediately preceding the trial. A one-way ANOVA showed no main effect of training day in this condition (F(15, 165) = 0.68, n.s.). Thus, an increase of the pupil size after the onset of the search display (Fig. 3(A)) can be said to reflect the mental effort invested in the task by the subjects.

## Discussion

If the improvement by perceptual learning leads to an automatization of cognitive and behavioral processes [5], the decrease of mental effort would be predicted. To examine this prediction, we measured pupil size while subjects trained for a visual conjunction search task. If pupil size reflects mental effort as suggested in previous studies [11]–[18], pupil size is expected to decrease as the search performance improves. We found that the reaction time needed in order to find a target monotonically decreases as learning proceeds. The performance improvement can be therefore represented by a typical learning curve (Fig. 1). Contrary to our prediction, the average pupil diameter during the visual search task increased rapidly in the early phase of learning (Figs. 3 to [Fig pone-0021973-g004] 5). Once it reached the maximum at the intermediate stage of the training course, it decreased to a level half of its maximum. It never returned to the level it was day before training. Thus, at least in the conjunction search task used, the improvement of the performance did not lead to a simple reduction of mental effort as suggested by Leonards et al [7]. Since the shape of the learning curve and pupil size function are different, an improvement of performance and increase of pupil size may reflect a different aspect of learning mechanism concerned.

What aspect of the learning mechanism does pupil size change reflect? One possibility comes from the observation that the subjective impression of the subjects was closely related to the pupil response. Verbal reports from some subjects roughly corresponded to their pupil response, though they were not systematically analyzed. For example, some subjects reported that they felt more tired after a session, especially when their response time was greatly improved. A large decrease in response time was observed at the early phase of training. Thus, though further studies are needed, pupil size might represent the subjective impression correlated with the amount of mental effort invested. Leonards et al [7] suggested a similar idea by measuring skin conductance response during a feature search task. They found that even when the behavioral performance to the visual search becomes parallel, which means the disappearance of the set-size effect by the training, the skin conductance level was still higher than that on the first day of training. They argued that parallel search does not have to be effortless and that subjects' feeling of tiredness is reflected in the skin conductance level.

Another possibility is that pupil response is related to the underlying attentional mechanisms. The fMRI study by Sigman et al [26] showed that the activity in the cortical area related to visual attention decreases after the learning of visual search. Yotsumoto et al [27] found that when visual search performance improves, the response of the V1 area decreases. The shape of the function of the BOLD response of the V1 area was inverted U shape, which is similar to the pupil size change shown in Fig. 4. It has been shown that pupil response is governed by activities in different visual areas [9]. Further studies are needed to clarify whether the V1 activity is one of the crucial signals for the modulation of pupil size.

In summary, we found that the amount of mental effort invested could not be simply correlated with behavioral performance in the visual perceptual learning. Autonomic nervous response, such as pupil response, is a promising candidate for clarifying a different aspect of learning other than the one the behavioral performance represents.

## Materials and Methods

### Ethics statement

The study reported here was reviewed and approved by the Research Ethic Committee of NTT Communication Science Laboratories. Written consent was obtained from all subjects after the nature and possible consequences of the studies were explained before starting the experiment.

### Subjects

Twelve subjects participated in the main experiment, and an additional twelve subjects participated in the experiment with the “passive condition”. All subjects were paid volunteers, who were naïve as to the purpose of the experiment. They had normal or corrected-to-normal vision.

### Apparatus

Visual stimuli were generated by MATLAB (MathWorks Inc.) with Psychophysics Toolbox extensions [28] [29] on an Intel-based PC (EPSON Endeavor MT7900), and displayed on a 21-in. RGB monitor (SONY GDM F520). The monitor frame rate was 120 Hz, with spatial resolution of 1024×768 pixels and 12 bit gray-level resolution. The monitor output was linearized (gamma corrected) under software control. For the experiment using luminance-varying stimuli, the space-averaged chromaticity (CIE1931) of the display was x = 0.31, y = 0.33. The room was darkened and shielded from light, with no other source of illumination present. Subjects observed the display with head position maintained by a chin and head rest. Patterns were viewed binocularly at a viewing distance of 57 cm.

The pupil diamter of the right eye of each subject was recorded with a ViewPoint EyeTracker 220 fps USB system (Arrington Research, Inc.) controlled by the same PC. Pupil diameter was sampled at 220 Hz by using a collection of MATLAB extensions, ViewPoint Toolbox, provided by Arrington Research, Inc.

### Procedures

Subjects conducted a visual conjunction search task, in which a target was defined by the combination of two visual attributes. The targets and distractors were Gabor patches, subtended 3.0 degrees of visual angle. In the example shown in Fig. 1, the target is located at the intersection of second row and column. In this case, only the target is oriented in the counter-clockwise direction, and its spatial frequency is high. The distractors had different combinations of orientation and spatial frequency. The spatial frequency was 1 or 2 cycles per degree, and the orientation was +2 (clockwise) or −2 degrees (counter-clockwise). These parameters were chosen based on the preliminary observation. The Michaelson luminance contrast of the Gabor pattern was 80%. The background of the visual search display was uniform gray, whose luminance was 42.0 cd/m^2^. The visual search display consisted of 20 deg×16 deg, and the stimuli were located among 16 positions on an imaginary grid composed of four rows and four columns. The set-size was 16 and the Gabor patches were located at the center of all imaginary grids.

In each trial, a target Gabor patch appeared for 1 sec on a uniform gray field. There were four types of target stimulus (two spatial frequencies and two orientations), which were randomly chosen for each trial. Then the uniform gray field with a small center fixation cross (1 deg×1 deg) appeared for 3 sec. Subjects were asked to fixate on the cross, and the average pupil diameter under fixation was used to normalize the pupil diameter obtained in the visual search task (see Fig. 3).

At a beep sound, the visual search display was appeared. Two small touch pads were placed close to the subject's right hand (All subjects were right-handed). Subjects were asked to tap one touch-pad if they found a target and tap the other if they found no target in the display. The position of the two touch-pads (right or left) was randomized between subjects. After the tapping, the uniform field was displayed for 5 sec, and subjects were asked to prepare for the next trial. No feedback was given at this time.

One experimental session consisted of eight trials, and each subject completed eight sessions per one day (total of 64 trials per day per subject). The type of the target, the order of target-present or target–absent trials, and the position of the target were randomized, but the number of target-present and target–absent trials were the same through all eight sessions. Subjects took 2 minutes of rest between sessions. After each session, the number of error trials was displayed on the screen to motivate subjects to try to make fewer errors.

Pupil diameter was recorded throughout each session. Analysis of pupil data was conducted offline. Eye blinking was removed from the data and interpolated by a spline transformation.

As described above, the average luminance of the display was the same throughout the experiment. Thus, the pupil light reflex could be minimized even though it occurs. To check the possibility of the pupil light reflex, we conducted a “passive” experiment, in which subjects only observed the visual search display for 4 sec with no serious search effort. Passive condition was run for one session (eight trials) per one day.
